# Plasma therapy cured a COVID‐19 patient with long duration of viral shedding for 49 days: The clinical features, laboratory tests, plasma therapy, and implications for public health management

**DOI:** 10.1002/mco2.2

**Published:** 2020-05-27

**Authors:** Li Tan, Xia Kang, Bo Zhang, Shangen Zheng, Bo Liu, Tiantian Yu, Fan Yang, Qiongshu Wang, Hongming Miao

**Affiliations:** ^1^ Department of Disease Control and Prevention General Hospital of Central Theater Command Wuhan People's Republic of China; ^2^ Department of Biochemistry and Molecular Biology Third Military Medical University (Army Medical University) Chongqing People's Republic of China; ^3^ Department of Infectious Disease General Hospital of Central Theater Command Wuhan People's Republic of China; ^4^ Department of Transfusion General Hospital of Central Theater Command Wuhan People's Republic of China; ^5^ Department of Laboratory No. 967 Hospital of PLA Dalian People's Republic of China

**Dear Editor,**

Coronavirus disease‐19 (COVID‐19), caused by severe acute respiratory syndrome coronavirus 2 (SARS‐CoV‐2), has become a threat to global health.^1^, ^2^, ^3^ The clinical spectrum in COVID‐19 patients presents diversety.^4^, ^5^ Viral shedding is a critical indicator for prognosis, and prolonged viral shedding always predicts a poor outcome.^5^, ^6^ In this report, however, we describe a special case of a family cluster with a mild type of COVID‐19. In one family member, the duration from illness onset has persisted for over 49 days till now, which is the longest duration of viral shedding in symptomatic patients as far as we know. Nevertheless, this case shows a mild infectivity and a better status comparing with most cases. Fortunately, treatment with plasma from recovered COVID‐19 patients efficiently cleared out the virus infection. This report describes the detailed epidemiologic and clinical information of this special patient and his close contacts, which might provide rationale for reasonable treatment and public health management.

## 1 A MODERATE PATIENT WITH LONG DURATION OF VIRAL SHEDDING

**FIGURE 1:**
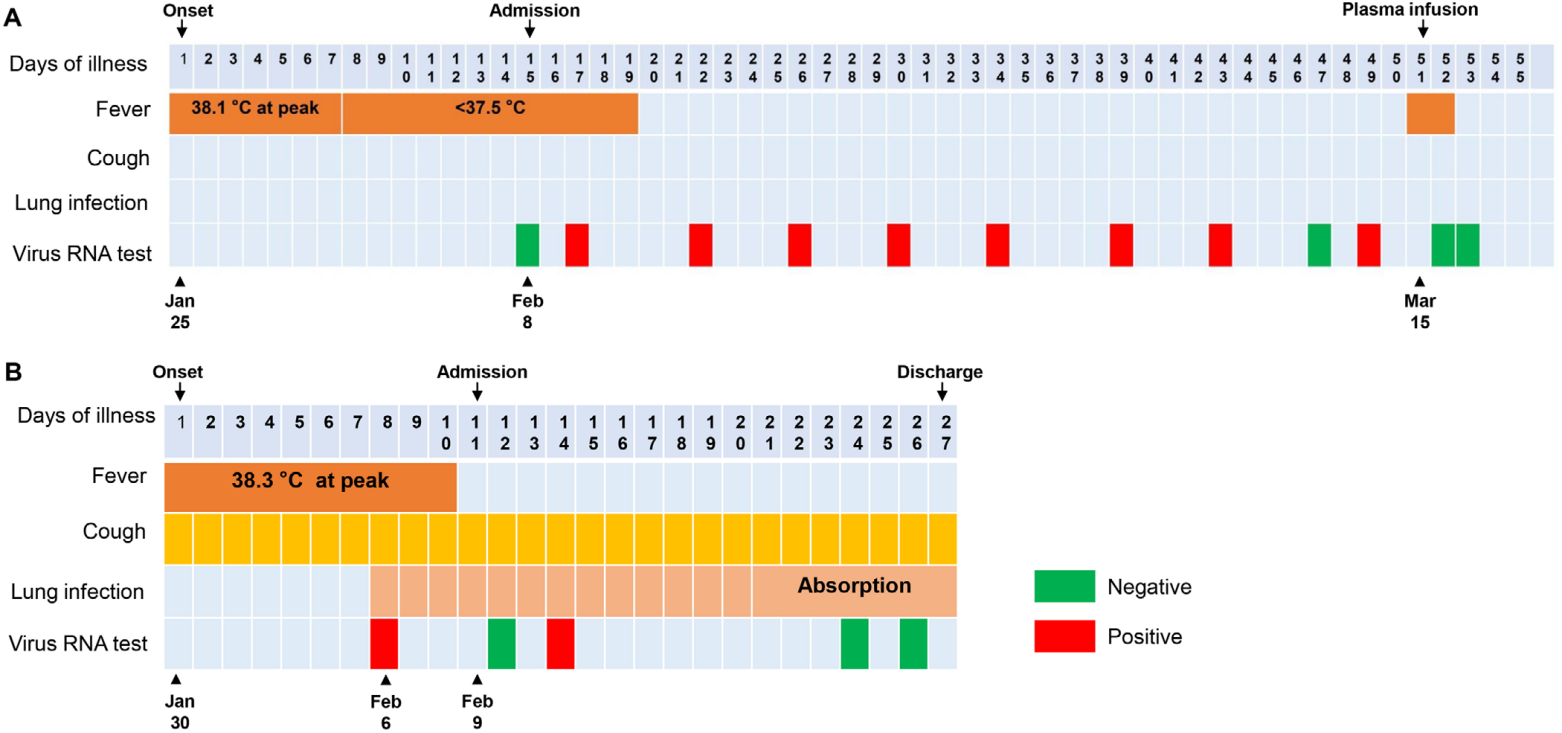
Dynamic assessment of symptoms, body temperatures and viral infection status of Case 1 (A) and Case 2 (B) from 25 January to 19 March 2020

A middle‐aged man (Case 1) visited our hospital to require SARS‐CoV‐2 tests on 8 February 2020 (Figure 1A). The patient stated that he had got an intermittent fever and continued for around 1 week since 25 January without other typical symptoms of COVID‐19, including chills, stuffiness, headache, dry cough, pharyngalgia, chest pain, shortness of breath, or diarrhea. The highest body temperature was 38.1°C. After taking antipyretics, Chinese traditional medicine, and antiviral medicine by himself, the temperature decreased to normal levels in 1 week. Because his close relative was confirmed to be infected with COVID‐19 1 day before, he asked for further examinations. On admission, the patient reported no subjective symptoms, whereas chest computed tomography (CT) scan showed infective signs in superior lobe of right lung and inferior lobes of bilateral lungs, with the laboratory tests in normal levels (Table S1). Nasopharyngeal swab specimens were obtained to test for influenza A and B and the results were negative. After admission, the patient received antiviral therapies and supportive care.

On days 2 through 5 of hospitalization, the patient presented with intermittent low fever, with the highest body temperature under 37.5°C and other vital signs stable (Figure 1A). A nucleic acid amplification test for COVID‐19 was positive (Figure 1A). Re‐examination of chest CT scan showed obvious resorption of the infection lesions of bilateral lungs. From hospital days 6 till now, the overall status of Case 1 remained stable. The patient presented with normal levels of vital signs including body temperature (Figure 1A). Nevertheless, COVID‐19 testing on specimens collected from oropharyngeal swabs on illness days 17, 22, 26, 30, 34, 39, 43, and 49 was positive and on illness day 47 was negative (Figure 1A). The detailed information on primary laboratory tests can be found in Figure S1. Noteworthily, the levels of blood lymphocytes, interleukin‐6, and procalcitonin have been recognized as indicators for disease severity and prognosis of COVID‐19 patients by us and others.^5^, ^7^ These three indexes were all normal and stable in this patient (Figure S2A‐C), whereas the viral load persisted in a high level as the severe or critical ill patients did (Figure S2D).

Given the prolonged infection, this patient received plasma infusion therapy. The plasma was collected from recovered patients with COVID‐19. On 15 March, Case 1 received 400 mL of plasma. The patient got fever in 4 h after infusion, with the body temperature up to 38.9°C, which we considered as a blood transfusion reaction. As expected, the body temperature became normal the next day. Notably, the viral tests of SARS‐CoV‐2 via oropharyngeal swabs on 16 and 17 March turned into negative, indicating that the plasma therapy might be a potent treatment for patients with COVID‐19.

## 2 A CLOSE CONTACT OF CASE 1 WITH MODERATE SYMPTOMS

An elderly woman (Case 2), a close relative of Case 1, visited our hospital on 9 February 2020. She complained an intermittent fever combined with occasional dry cough for around 10 days. Before visit, the highest body temperature was 38.3°C. On 6 February, chest CT showed bilateral lung infective signs in this patient. Laboratory tests revealed an elevation of neutrophil percentage (82.3%) and C‐reactive protein (CRP) level (61 mg/L). The patient was prescribed antiviral medicines and Chinese traditional medicines. However, the fever was not absolutely controlled after receiving these treatments for 3 days (Figure 1B and Table S1).

After admission, the vital signs were stable. Re‐examination of chest CT scan revealed that exudative lesions in bilateral lungs became worse on hospital days 5. However, on hospital days 11, the lung infection attenuated significantly. For laboratory tests, the percentage of neutrophils (70.70%) and CRP level (9.81 mg/L) on hospital days 2 dramatically decreased comparing with those on 5 days before. SARS‐CoV‐2 tests presented positive on specimens collected from oropharyngeal swabs on hospital days 4, but it turned into negative on hospital days 14 and 16. Then the patient was permitted to discharge from hospital (Figure 1B and Table S1).

## 3 LONG DURATION OF VIRAL SHEDDING MIGHT BE ASSOCIATED WITH VIRUS TYPES

It was reported that the median duration of viral shedding was 20.0 days from disease onset and the longest was 37 days.^5^ There were no significant differences between severe patients (19 days) and critical patients (24 days).^5^ In our report, the duration of viral shedding from illness onset in Case 1 has persisted for 49 days, which has been the longest in ever reported in symptomatic patients.

Previous studies indicated the level and duration of viral shedding is a critical indicator to access the risk of transmission and to guide the isolation of patients as well as predicting the prognosis.^5^, ^6^ In viral infection, prolonged viral shedding was associated with inferior outcome.^6^ Interestingly, contrary to the conclusions above, we here reported that one of the nonsevere cases has the longest duration of viral shedding. The Case 1 only got moderate fever initially and the body temperature rapidly decreased into normal levels without any respiratory failure. Although Reverse Transcription Polymerase Chain Reaction analysis showed virus was not eliminated, the symptoms and signs have been largely stable after admission.

Notably, epidemiological survey revealed that besides his close relative, Case 1 closely contacted with another five people before admission. One contact also got fever for a short term but the test for SARS‐CoV‐2 showed negative. The other four contacts without any symptoms were also negative in SARS‐CoV‐2 tests.

These clues together indicated that the SARS‐CoV‐2 virus in Case 1 and Case 2 might be a mild subtype, because Tang et al reported that the virus evolved into two major types (namely, L subtype and S subtype).^8^ Currently, few studies focused on identifying the clinical features between these two subtypes. It is worthy for us to further analyze the mRNA sequence of the virus type isolated from Case 1, which will help us to distinguish potential mild patients.

## 4 IMPLICATIONS FOR PUBLIC HEALTH MANAGEMENT

Although evidences collected from these two cases indicate a better prognosis, patients with long duration of viral shedding in mild type are likely to be neglected in crowd and may persist infecting surroundings and cause a new outbreak. The viral tests of Case 1 once changed into negative on illness days 47. However, it reversed into positive on illness days 49 (Figure 1A). It is likely that the virus and the host got a dynamic balance. Although the viral duplication was suppressed, immune cells cannot clear out the virus. The Case 1 may tend to be a chronic infected case without infusion treatment. We wondered how many patients involved in this situation. One important question is whether and how long this kind of patients keeps infective. The other important question is whether the “chronic infected patients” will infect through new route of transmission, such as sexual transmission. Moreover, due to the high mutation rate of retrovirus, we should keep close eyes on the status of this kind of patients and the infective ability.

In conclusion, we reported the clinical features of a special family cluster case, of which one member has the longest duration of viral shedding in current reports. Our report points out plasma infusion might be an efficient therapy for COVID‐19 patients with long duration of viral shedding and provides valuable information for public health management.

## CONFLICT OF INTEREST

The authors declare no conflict of interest.

## AUTHOR CONTRIBUTIONS

Li Tan, Qiongshu Wang, and Bo Zhang contributed to data collection and summary. Li Tan, Shangen Zheng, Bo Liu, Tiantian Yu, and Fan Yang participated in discussion. Xia Kang and Hongming Miao wrote this draft. Hongming Miao designed and revised this manuscript.

## Supporting information

Supporting Information.
