# Supplementary material for: Plasma therapy cured a COVID‐19 patient with long duration of viral shedding for 49 days: The clinical features, laboratory tests, plasma therapy, and implications for public health management
Source: MedComm (2020). 2020 May 27;1(1):77–80. doi: 10.1002/mco2.2 (PMC7262084; doi:10.1002/mco2.2)
Supplement: Supplementary file 1 — Supporting Information. [file MCO2-1-77-s001.docx]

Supplementary Materials for

Plasma therapy cured a COVID-19 patient with long duration of viral shedding for 49 days

Li Tan^#^, Xia Kang^#^, Bo Zhang, Shangen Zheng, Bo Liu, Tiantian Yu, Fan Yang, Qiongshu Wang^*^, Hongming Miao^*^

Correspondence to: hongmingmiao@sina.com; whzyywqs@163.com

**This PDF file includes:**

Methods and References

Figure S1 to S2

Table S1

**Methods**

**Disease classification of COVID-19**

All cases were diagnosed and classified according to the New Coronavirus Pneumonia Diagnosis Program (5th edition)^1^ published by the National Health Commission of China. Clinical manifestations consist of four categories, mild, moderate, severe and critical. The mild clinical symptoms were mild with no pulmonary inflammation on imaging or without symptoms of respiratory infections. The moderate is the overwhelming majority, showing symptoms of respiratory infections such as fever, cough, and sputum, and pulmonary inflammation on imaging; when symptoms of dyspnea appear, including any of the following: shortness of breath, RR ≥ 30bpm, blood oxygen saturation ≤ 93% (at rest), PaO_2_ / FiO_2_ ≤ 300 mmHg, or pulmonary inflammation that progresses significantly within 24 to 48 hours> 50%, it was classified as severe; respiratory failure, shock, and organ failures that require intensive care were critically ill.

**Patient information**

In this study, all cases were taken from the General Hospital of Central Theater Command (Wuhan, Hubei province, People’s Republic of China), one of designated hospital for the COVID-19 by local authority. This study was approved by the Ethics Committee of the hospital. All subjects signed informed consent forms at admission to hospital. 130 patients including the discharged and dead in our hospital between January 14, 2020 and March 19, 2020 were investigated and their clinical indexes were used as references of a hospitalized patient (Case 1), who would be introduced in detail in this study. There were 96 cases of moderate type, 19 cases of severe type and 15 cases of critically ill type (all died). Another patient Case 2, the close relative of Case 1, was also followed. All patients were confirmed by viral detections using quantitative RT-PCR^2^, which ruled out infection by other respiratory viruses such as influenza virus A, influenza virus B, coxsackie virus, respiratory syncytial virus, parainfluenza virus and enterovirus by the same time.

**Data collection**

In this study, the basic information, clinical symptoms, complete blood count, coagulation profile, and serum biochemical test (including renal and liver function, creatine kinase, lactate dehydrogenase, and electrolytes) and disease outcome of all included patients were collected. The epidemiological data of the investigated patient Case 1 was also collected.

**Statistical methods**

In this study, GraphPad 6.0 software was used for data statistics and mapping. All the data were displayed descriptively.

**References**

1. Zu ZY, Jiang MD, Xu PP, et al. Coronavirus Disease 2019 (COVID-19): A Perspective from China. Radiology 2020:200490.

2. Corman VM, Landt O, Kaiser M, et al. Detection of 2019 novel coronavirus (2019-nCoV) by real-time RT-PCR. Euro Surveill 2020;25.


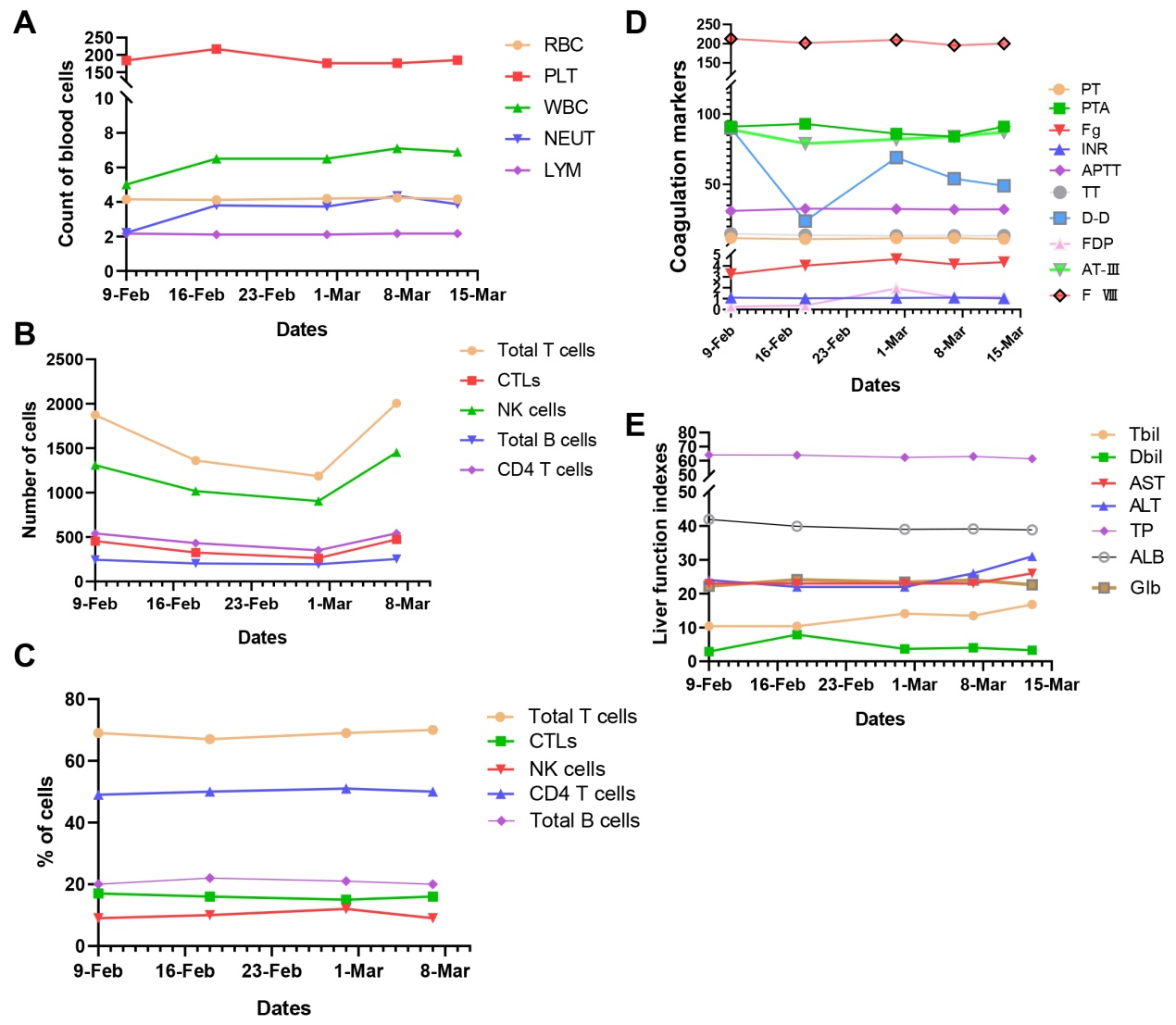


**Figure S1 Dynamic tests of blood indexes in Case 1 after admission**

(A) The count of blood cells. RBC (×10^12^/L), PLT (×10^9^/L), WBC (×10^9^/L) , NEUT (×10^9^/L) and LYM (×10^9^/L)

(B) The count of peripheral lymphocytes and NK cells. Total T cells (/ul), CTLs (/ul), NK cells (/ul),Total B cells (/ul) and CD4 T cells (/ul)

(C) The percentages of peripheral lymphocytes and NK cells

(D) The tests for coagulation indicators. PT (s), PTA (%), Fg (g/L), APTT (s), TT (s), D-D (ng/ml), FDP (μg/ml), AT-Ⅲ (%), F Ⅷ (%)

(E) The tests of liver function indicators. Tbil (μmol/L), Dbil (μmol/L), AST (U/L), ALT (U/L), TP (g/L), ALB (g/L), GLB (g/L)

RBC, red blood cells; PLT, platelets; WBC, white blood cells; NEUT, neutrophils; LYM, lymphocytes; CTLs, cytotoxic T lymphocytes; NK cells, natural killer cells; PT, prothrombin time; PTA, prothrombin time activity; Fg, fibrinogen; INR, international normalized ratio; APTT, activated partial thromboplastin time; TT, thrombin time; D-D, d-dimer; FDP, fibrin/fibrinogen degradation products; AT Ⅲ, antithrombin Ⅲ; Tbil, total bilirubin; Dbil, direct bilirubin; AST, aspartate transaminase; ALT, glutamic-pyruvic transaminase; TP, total protein; ALB, albumin; Glb, globulin


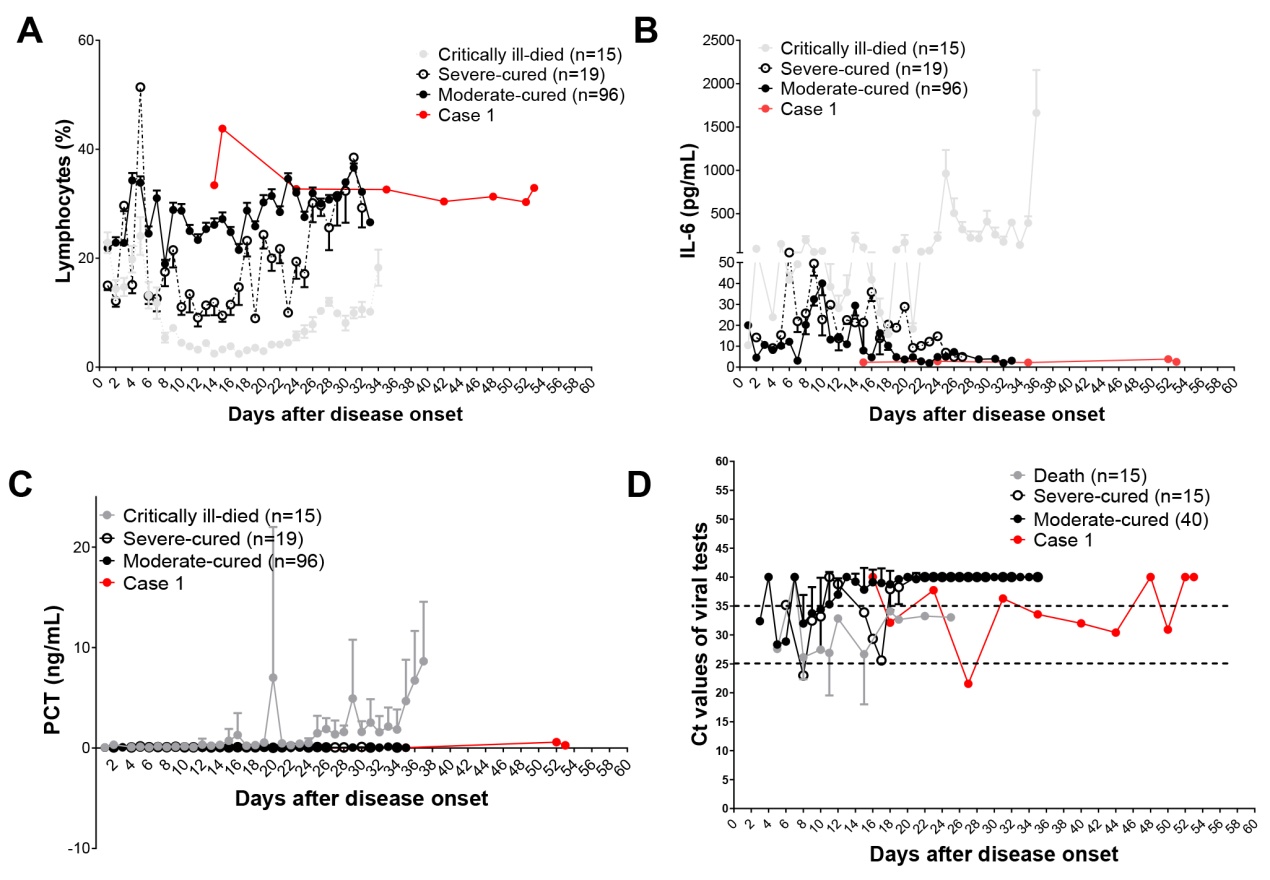


**Figure S2** Dynamic changes of lymphocyte percentage (A), IL-6 levels (B) and PCT levels (C) in peripheral blood and Ct values of viral tests (D) in the moderate-cured, severe-cured and critically ill patients and Case 1 with COVID-19. The parameters of cured or dead patients were used as references of Case 1. Severe-cured, severe type of patients with a cured outcome; Moderate-cured, Moderate type of cases with a cured outcome; IL-6, interleukin-6; PCT, procalcitonin

**Table S1.** Basic and clinical information of two COVID-19 patients on admission

| **Items** | **Case 1** | **Case 2** |
| --- | --- | --- |
| **Demographics & clinical characteristics** | | |
| Age range (years) | 40-50 | 70-80 |
| Sex | male | female |
| ABO | AB | NA |
| Smoking | N | N |
| Comorbidity | N | Rheumatoid arthritis |
| Respiratory rate (on admission) | 14 | 20 |
| Pulse (on admission) | 88 | 82 |
| Systolic blood pressure (on admission) | 112 | 138 |
| Diastolic blood pressure (on admission) | 68 | 69 |
| Fever | Y | Y |
| Cough | N | Y |
| Diarrhoea | N | N |
| Nausea | N | N |
| Chest pain | N | N |
| Disease severity status | Moderate | Moderate |
| Time from illness onset to hospital admission (days) | 15 | 10 |
| **Laboratory findings (on admission)** | | |
| Red blood cell count (×10^12^/L) | 4.56 | NA |
| Hemoglobin (g/L) | 137 | NA |
| Platelet count (×10^9^/L) | 222 | NA |
| White blood cell count (×10^9^/L) | 6.6 | 6.95 |
| Lymphocyte count (×10^9^/L) | 2.19 | 1.21 |
| % Lymphocyte | 33.4 | 17.40 |
| Neutrophil count (×10^9^/L) | 3.61 | 4.91 |
| % Neutrophil | 55.1 | 70.70 |
| Monocyte count (×10^9^/L) | 0.68 | NA |
| % Monocyte | 10.4 | NA |
| Albumin (g/L) | 41.9 | 38.4 |
| ALT (U/L) | 24 | NA |
| Creatinine (μmol/L) | 79 | 89.8 |
| Uric acid | 232 | NA |
| Prothrombin time | 11.8 | NA |
| D-dimer | 90 | NA |
| IL-6 (pg/mL) | 2.3 | NA |
| **Imaging features (on admission)** | | |
| Bilateral pulmonary infiltration | Y | Y |

N, No; Y, Yes; NA = Not available
